# Clinical Challenges and Management Strategies in Pulmonary Large-Cell Neuroendocrine Carcinoma

**DOI:** 10.7759/cureus.63781

**Published:** 2024-07-03

**Authors:** Axle D Untalan, FNU Arty, Mahrukh A Khan, Vishakha Sirpal, Shazia M Shah

**Affiliations:** 1 Internal Medicine, Rutgers Health/Monmouth Medical Center, Long Branch, USA

**Keywords:** bronchial stent, vats, lung resection, neuroendocrine carcinoma, lung cancer, lcnec

## Abstract

Pulmonary large-cell neuroendocrine carcinoma (LCNEC) is a rare but aggressive malignancy of the lung. Its nonspecific presentation and propensity for severe disease at the time of diagnosis create challenges in treatment. We report a case of an asymptomatic 61-year-old female who was incidentally found to have a pulmonary nodule after a fall. Upon further workup, she was found to have an aggressive LCNEC. The patient underwent a robotic-assisted left upper lobectomy, which was complicated by left lower lobe bronchus kinking and post-obstructive atelectasis, warranting further management by thoracic surgery. The patient additionally underwent video-assisted thoracoscopic surgery (VATS), open thoracotomy, pneumopexy, and bronchial stenting. This case highlights the need for strategies for early detection in at-risk populations. A multidisciplinary approach, which may involve both medical and surgical subspecialties, is essential in the management of this complex disease from the time of diagnosis to follow-up postoperatively to achieve the best clinical outcome.

## Introduction

Pulmonary large-cell neuroendocrine carcinoma (LCNEC) presents a formidable clinical challenge due to its rarity, aggressive nature, and poor prognosis, accounting for a mere 2.5%-3% of all lung cancer cases [1,2]. LCNEC shares characteristics of both small-cell lung cancer (SCLC) and non-small-cell lung cancer (NSCLC), confounding both diagnoses and treatment strategies. It typically affects heavy-smoking White males approximately 65 years old [3]. LCNEC often evades detection until advanced stages, heralded by nonspecific symptoms or incidental findings. In this case study, we delve into the diagnostic journey and therapeutic considerations for a 61-year-old female with a significant smoking history who was incidentally discovered to have a pulmonary nodule while hospitalized for a rib fracture following a fall. Through this exploration, we aim to elucidate the complexities surrounding LCNEC management and underscore the importance of a multidisciplinary approach to optimizing patient outcomes.

## Case presentation

**Figure 1:**
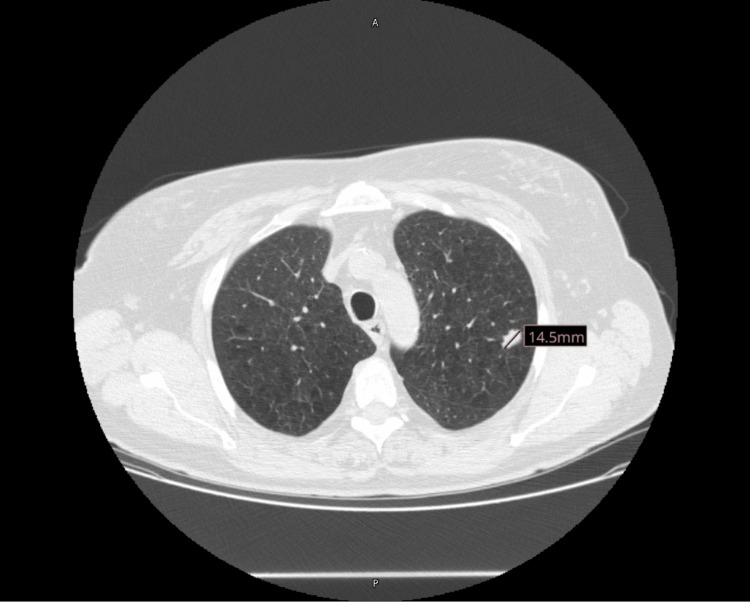
The initial chest CT scan shows a 1.4 cm spiculated left upper lobe nodule.

A 61-year-old female with a medical history significant for chronic obstructive pulmonary disease (COPD), nicotine dependence, and rheumatoid arthritis had a fall resulting in multiple rib fractures on the right. This was confirmed by a chest radiograph revealing an incidental left upper lobe pulmonary nodule. The chest CT scan conducted on June 14, 2022, was remarkable for minimally displaced rib fractures on the 8th, 9th, 10th, and 11th ribs on the right. Additionally, a 1.4 cm spiculated nodule was observed in the left upper lobe (Figure 1), corresponding to a standardized uptake value (SUV) of 1.9 on a positron emission tomography (PET) scan conducted on July 7, 2022. No other areas of fluorodeoxyglucose (FDG) uptake were observed.

**Figure 2:**
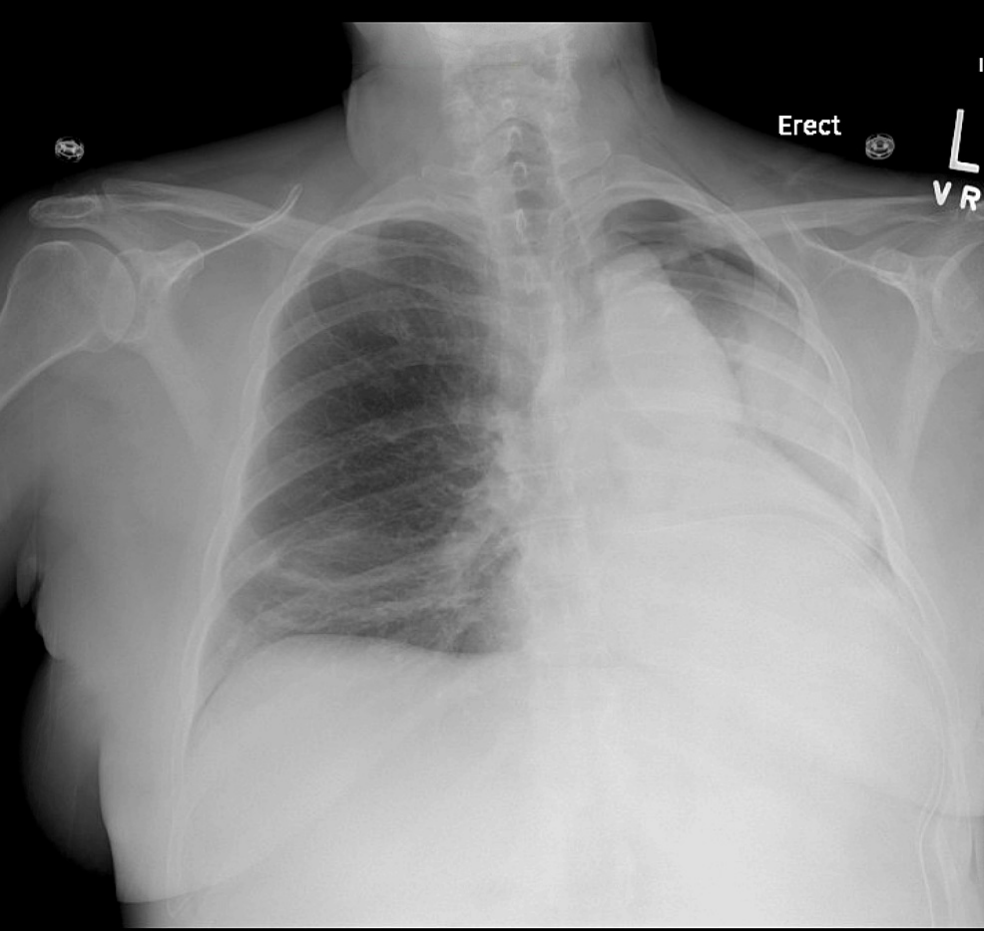
The chest X-ray reveals a large left-sided pneumothorax and total left lung opacification, which is associated with a heart and mediastinal shift to the left.

The patient was evaluated by thoracic surgery and was scheduled for lung nodule resection on August 23, 2022. A bronchoscopy revealed that friable lymph nodes surrounded the nodule. The left upper lobe was also noted to be severely emphysematous, requiring robotic-assisted left upper lobectomy and lymph node dissection, which were both performed without any immediate postoperative complications. A postoperative chest X-ray revealed good air expansion with a chest tube in the appropriate position. A sequential chest X-ray on August 24, 2022, was remarkable for a large left-sided pneumothorax and total left lung opacification associated with heart and mediastinal shift to the left, compatible with near-total left lung volume loss/atelectasis, possibly postobstructive in nature. The pneumothorax was presumed to be ex vacuo in this setting (Figure 2).

**Figure 3:**
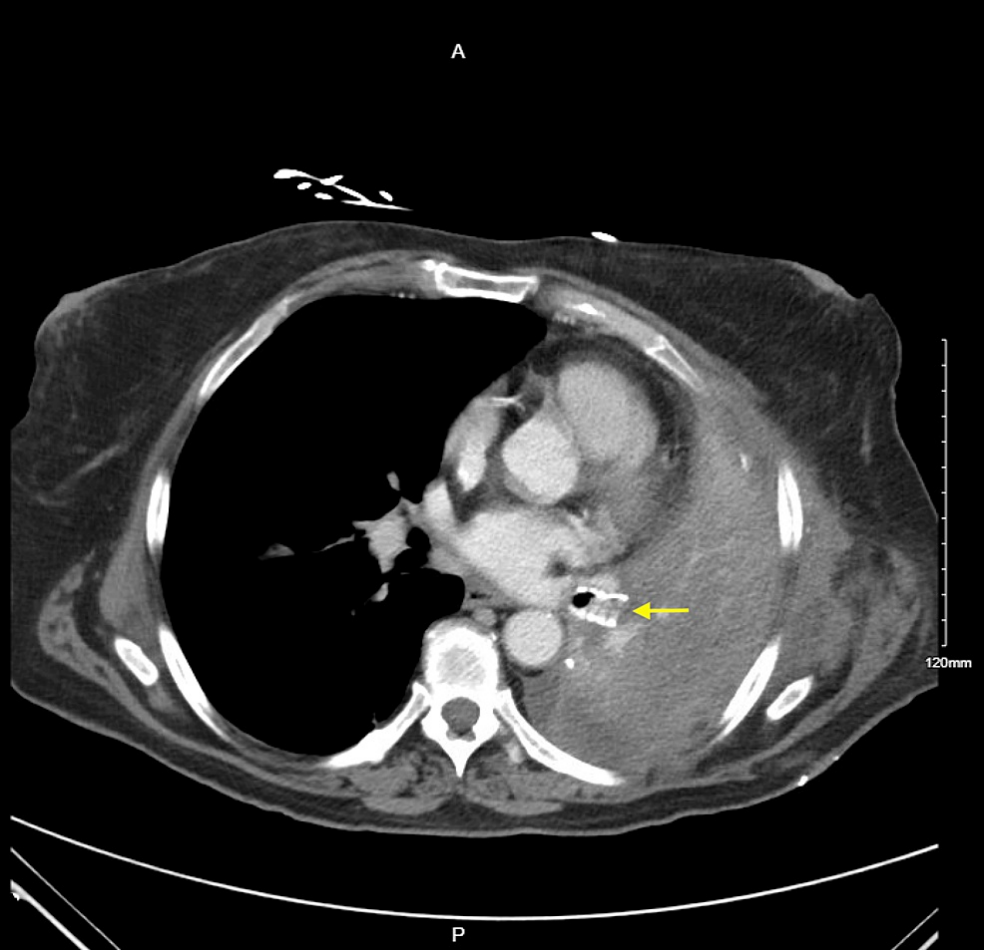
Postoperative CT chest showing the bronchial stent in place (yellow arrow).

The mucus plugging noted during a repeat bronchoscopy was aspirated. The X-ray findings were suspicious of left bronchus angulation, so the decision was made to keep the patient intubated, transfer to the ICU, and administer steroids to reduce inflammation. Lactic acidosis (level 2.7) was noted and caused concern for sepsis, prompting the collection of blood and urine cultures and initiating empiric antibiotics with cefepime. An overnight chest X-ray revealed left lung volume loss, and the patient was immediately taken to the operating room for left video-assisted thoracoscopic surgery (VATS) and open thoracotomy, which revealed kinking of the left lower lobe bronchus. The lung was repositioned, and a pneumopexy was performed in addition to alloderm placement for apical pleural tenting. Repeat bronchoscopy on August 25, 2022, revealed a narrowed left bronchus requiring a left inferior bronchus stent insertion by thoracic surgery on August 26, 2022. The patient tolerated the procedure well and was successfully extubated. A repeat CT of the chest done on September 1, 2022, showed the patient's status following the left upper lobectomy. The bronchial stent was seen in place (Figure 3).

**Figure 4:**
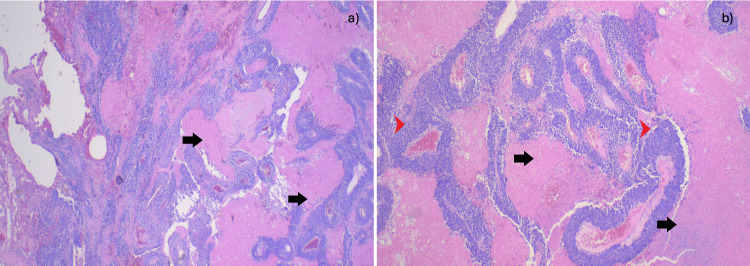
(a) At 2x magnification, nests of tumor cells are visible, with palisading extensive necrosis. The black arrows point to extensive necrosis. (b) At 4x magnification, nests of medium-to-large atypical ovoid cells are observed, with coarse chromatin with extensive necrosis in a palisading pattern. The black arrows point to necrosis. The red arrowheads point to nests of palisading cells.

**Figure 5:**
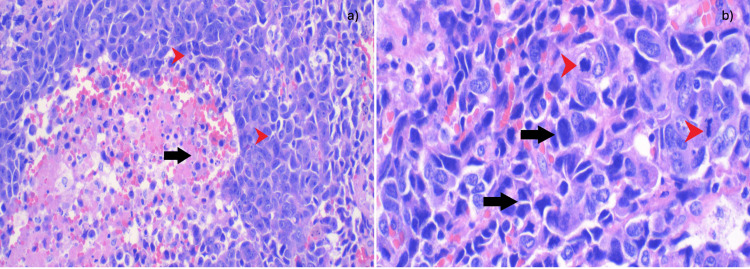
(a) 20x magnification: medium-to-large atypical ovoid cells with coarse chromatin (black arrows). Apoptotic bodies and necrotic cells can also be seen (red arrowheads). (b) 40x magnification: medium-to-large atypical ovoid cells with coarse chromatin (black arrows). Abnormal mitosis can be seen (red arrowheads).

**Figure 6:**
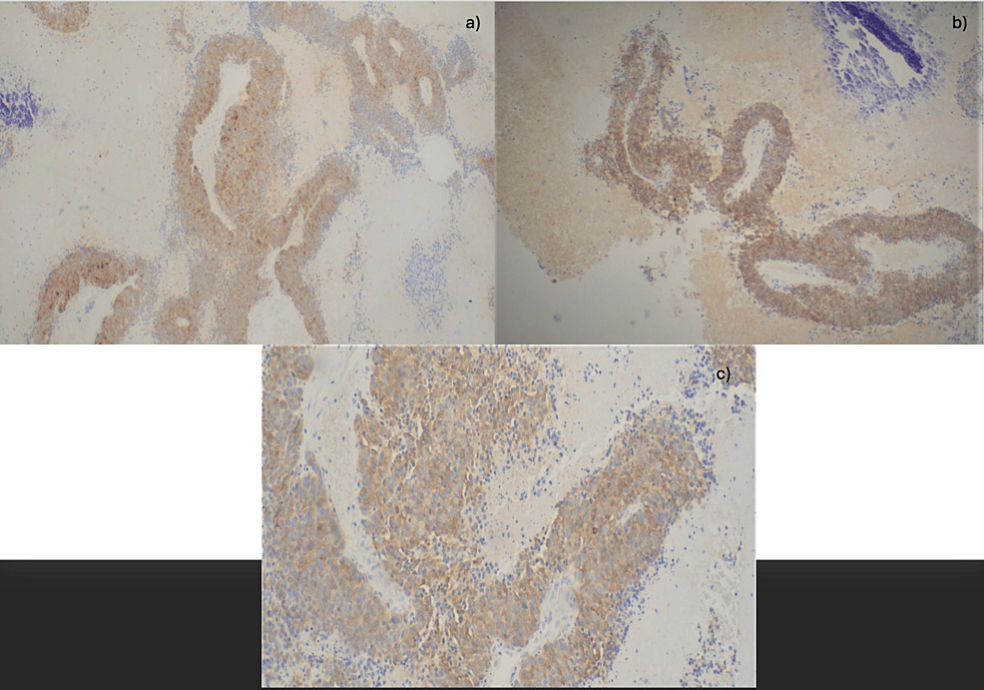
Brown staining pattern of tumor cells can be identified. (a) 4x magnification: positive for synaptophysin. (b) 4x magnification: positive for CD56. (c) 10x magnification: positive for CD117.

The pathology report confirmed limited but high-grade large cell neuroendocrine neoplasm with extensive necrosis (Figures 4, 5), aggressive with a Ki-67 proliferation index of 80% and mitotic rate of 20/mm^2^. Immunohistochemical stains were performed with adequate controls. The tumor cells were positive for CD56, synaptophysin, CD117 (Figure 6), TTF-1, and chromogranin, while negative for Napsin A, CK5/6, and p63.

A plan for outpatient adjuvant chemotherapy with etoposide and cisplatin was instated for her limited but aggressive neuroendocrine tumor (T1BN0, stage 1) by hematology-oncology. Blood, urine, and tracheal aspirate cultures showed no growth. The patient was successfully weaned off supplemental oxygen and was discharged home on September 4, 2022. The bronchial stent was removed by thoracic surgery on September 9, 2022. The patient has been seen regularly for follow-up at six-month intervals and has had no further pulmonary issues.

## Discussion

LCNEC is a rare and formidable malignancy, constituting merely 2.5%-3% of all lung cancer cases [3,4]. Its pathology bridges the features of both SCLC and NSCLC, posing diagnostic and therapeutic challenges [3,4]. Predominantly afflicting heavy-smoking White males, LCNEC tends to emerge around the age of 65 [3,4].

Symptomatically elusive, LCNEC often remains asymptomatic or manifests with nonspecific complaints such as cough, hemoptysis, and chest discomfort [5]. Notably, its diagnosis frequently occurs incidentally, with metastases already disseminated, commonly to the liver, bone, brain, adrenal gland, pleura, and extra-thoracic lymph nodes [3,6]. In our case, a 61-year-old female with a significant smoking history was incidentally discovered to harbor a lung nodule while hospitalized for a rib fracture following a fall.

Radiologically, LCNEC presents with distinctive features, including irregular margins, emphysema, notching, and cavity formation [7]. Our patient exhibited an incidental left upper lobe pulmonary nodule, with further CT assessment revealing pertinent details, including size and metabolic activity on PET-CT.

Histopathological confirmation through biopsy is pivotal prior to treatment initiation. The 2021 WHO Classification delineates stringent criteria for LCNEC diagnosis, comprising neuroendocrine and non-small-cell cytological features alongside immunohistochemical markers [4,7]. CD56, chromogranin A, and synaptophysin are sensitive and specific markers for neuroendocrine cancers [8,9]. Our patient's bronchoscopy revealed necrotic nodules with corroborative histopathological features confirming LCNEC.

Therapeutic strategies for LCNEC remain heterogeneous due to its rarity and aggressive nature [10]. Surgical resection is favored for early-stage disease, supplemented by adjuvant chemotherapy to mitigate the poor prognosis. To our knowledge, bronchial kinking is not known to be a characteristic finding of LCNEC, but it is a relatively common postoperative change that can be expected following lung lobectomy due to the displacement of the remaining lobes, elevation of the diaphragm, a shift of the mediastinal organs, and narrowing of the intercostal space [10]. Chemoradiotherapy serves as an alternative for inoperable cases or those with locally advanced diseases [7,11]. For advanced stages, platinum-based chemotherapy regimens, often in conjunction with other agents such as etoposide or irinotecan, constitute the mainstay. Immunotherapy, though promising, necessitates further elucidation in LCNEC management [7,11]. Our patient's treatment plan involves outpatient adjuvant chemotherapy with etoposide and cisplatin, tailored to address her limited but aggressive neuroendocrine tumor at an early stage (T1BN0, stage 1).

In summary, LCNEC poses a diagnostic and therapeutic conundrum due to its rarity and aggressive behavior. A multidisciplinary approach, integrating surgical, chemotherapeutic, and potentially immunotherapeutic modalities, is imperative to improve outcomes in this challenging disease paradigm.

## Conclusions

This report presents the complicated course of a patient found to have a pulmonary LCNEC after a workup for an incidental left lung mass. Multiple factors, including the absence of symptoms and the need for tissue sampling for pathologic assessment, make early diagnosis challenging. The patient’s course post-lobectomy was complicated by bronchial kinking and atelectasis, requiring further surgical interventions. This case highlights the importance of a multidisciplinary approach in managing this complex disease from the time of diagnosis to follow-up postoperatively to achieve the best clinical outcome.
